# A Note on Chronic Frontal Sinusitis

**Published:** 1899-11-04

**Authors:** 


### A NOTE ON CHRONIC FRONTAL SINUSITIS.

Attention may be drawn to a point which arose in the discussion at Portsmouth on chronic suppuration of the frontal sinuses, namely, that the association of polypi in the nose with creamy pus indicates suppuration in one or other of the sinuses, and, further, that the polypi are secondary to the suppuration, so that in such cases the mere removal of the polypi is not sufficent. Having removed the polypi and the granulations from the middle meatus, the pus can be seen as a creamy head, and its source must be ascertained. And here it is to be borne in mind that the affection of the maxillary may be secondary to that of the frontal, the pus from the latter flowing down into the former.

